# Endowment of the Atlanta Medical College

**Published:** 1858-01

**Authors:** 


### EDITORIAL AND MISCELLANEOUS.

### Endowment of the Atlanta Medical College.

We are happy to have it to announce to the many kind friends who have taken an interest in the welfare of the Atlanta Medical College, that the Legislature of Georgia, at its recent session, made a handsome appropriation for its benefit.

This liberality upon the part of the State will place the institution upon a still more firm and impregnable basis, by relieving it entirely from embarrassment, and furnishing the means of completing the building according to the original design, which will add greatly to its external appearance, and make it an architectural ornament to the city.

When we recollect what has been accomplished in three years by those who have built up this institution, in the face of the most discouraging circumstances, we feel that it is difficult to put a limit upon its success, provided we have a continuance of the same energetic and determined purpose which has heretofore characterized its founders. That we shall have this, and even more, we hesitate not to say. The recent munificence of the State will only serve as an additional stimulus to the energies of the Faculty, and we feel authorized in asserting that they have no lower ambition than to furnish advantages to the medical student equal to those to be found in any institution in the land ; and not only this, but to establish the *highest standard of requisitions for graduation* which shall be adopted and *conformed to* by the medical colleges of the United States.

Those who have been guilty of the contemptible effort to produce the impression that *the season of the year* at which the lectures were delivered, was intended to lower the standard, may yet learn that they are behind those who control the Atlanta Medical College, in the race for the elevation of Medical education.

The *catachetical* plan of teaching which has been introduced into this College, in addition to the old plan of lectures, has already contributed to the more thorough instruction of the students who have been in attendance here, so much so, indeed, as to have superseded the demand for what has been heretofore considered an indispensable appendage to a Medical College, the *private drill or Quiz*, for the *preparation* (not instruction) of those who were to become candidates for graduation. And as suggestions upon the subject of medical education seem to be the order of the day, we take the liberty of urging upon those who are directing their attention to this matter, the adoption of the plan embracing regular and systematic recitations previous to each lecture, upon that which has preceded it from the same professor, the advantages of this plan being so perfectly manifest to those who have witnessed it, that they are forced to the conclusion that the student is really more benefitted by the recitation and accompanying recapitulation than by the formal lecture upon which they are based.

We then say to those who have taken medical education *just now* under their peculiar guardianship, "*come on*," and "show your faith by your works," and not attempt by vain boasting, supercilious sneers, or yet more reprehensible conduct, to bring your competitors into disrepute by pretensions to which you have no just claim.

While the recent endowment of the College has been mainly due to the indefatigable exertions of our Senator and Representative in the Legislature, (Messrs. WHITAKER and WESTMORELAND,) in presenting its merits, we yet owe a debt of gratitude to a large number of the most prominent and intelligent members of both Houses who investigated and acknowledged our claims to aid, and who have manifested an interest in the enterprise which shall nerve every energy to the upbuilding of an institution that shall be an honor to the State, and in this way make some fit return to the Legislature of 1857, towards whose intelligence and appreciation of the true interests of the State, we feel in a particularly complimentary mood.

The Atlanta Medical College is emphatically and peculiarly a *Southern* institution, from the fact that its sessions are held *only in the summer*, and on this account is cut off entirely from Northern patronage, and thrown wholly upon *home* resources for support, which, however, are amply sufficient to meet the largest desires, if its true position is appreciated. Occupying as it does the only point in the Southern country where summer medical teaching can be successfully conducted, —being one of the most pleasant and healthy localities in the whole United States, (the summer being far more pleasant than many degrees farther north, from the great elevation of the country,)—never having been visited by any of those fatal epidemics which scourge more or less almost every other city of any importance in the South, and possessed of a centrality, and especially an accessibility, unsurpassed, combined with a cheapness of living scarcely found anywhere in a city of the same size, it can not be otherwise than that the institution must be permanently sustained and become eminently prosperous, if the people of the South are true to their own most manifest interests. From the remarkable progress of the institution thus far, we have good reason to conclude that it was a *desideratum*, to furnish our Southern young men facilities for the pursuit of medical knowledge during the summer months upon their own soil, in a pleasant, accessible and perfectly healthy locality; in vindication of the truth of which inference, we would state in conclusion, for the benefit of those who have not been made acquainted with the statistics, (thinking it possible that this number of our Journal will fall into the hands of many who are not fully acquainted with its history,) that the College has been in operation three years; the first year numbering 79, the second year 105, and the third 125 matriculates, with the prospect (notwithstanding the great financial embarrassment of the country,) of an increased class at the next session.

